# *Staphylococcus aureus* Lpl Lipoproteins Delay G2/M Phase Transition in HeLa Cells

**DOI:** 10.3389/fcimb.2016.00201

**Published:** 2016-12-27

**Authors:** Minh-Thu Nguyen, Martine Deplanche, Mulugeta Nega, Yves Le Loir, Loulou Peisl, Friedrich Götz, Nadia Berkova

**Affiliations:** ^1^Microbial Genetics, Interfaculty Institute of Microbiology and Infection Medicine Tübingen, University of TübingenTübingen, Germany; ^2^Institut National de la Recherche Agronomique (INRA), UMR1253 Science & Technologie du Lait & de l'Oeuf (STLO)Rennes, France

**Keywords:** lipoproteins, lipopeptides, G2/M phase, cell cycle, *Staphylococcus aureus*

## Abstract

The cell cycle is an ordered set of events, leading to cell growth and division into two daughter cells. The eukaryotic cell cycle consists of interphase (G_1_, S, and G_2_ phases), followed by the mitotic phase and G_0_ phase. Many bacterial pathogens secrete cyclomodulins that interfere with the host cell cycle. In *Staphylococcus aureus* four cyclomodulins have been described so far that all represent toxins and are secreted into the culture supernatant. Here we show that the membrane-anchored lipoprotein-like proteins (Lpl), encoded on a genomic island called νSaα, interact with the cell cycle of HeLa cells. By comparing wild type and *lpl* deletion mutant it turned out that the *lpl* cluster is causative for the G2/M phase transition delay and also contributes to increased invasion frequency. The lipoprotein Lpl1, a representative of the *lpl* cluster, also caused G2/M phase transition delay. Interestingly, the lipid modification, which is essential for TLR2 signaling and activation of the immune system, is not necessary for cyclomodulin activity. Unlike the other staphylococcal cyclomodulins Lpl1 shows no cytotoxicity even at high concentrations. As all Lpl proteins are highly conserved there might be a common function that is accentuated by their multiplicity in a tandem gene cluster. The cell surface localized Lpls' suggests a correlation between G2/M phase transition delay and host cell invasion.

## Introduction

Host-pathogen interaction is a complex process whose outcome depends on one hand on sophisticated strategies by pathogens to avoid the immune surveillance and on the other hand on the protective reaction of the infected host (Casadevall and Pirofski, 2000). Pathogens can target various host cell pathways such as apoptosis, cytoskeletal organization, chromatin organization and others (Dumoux et al., 2015; Rolando et al., 2015; Olsen and Hajishengallis, 2016). In the past decade special attention was paid to the group of bacterial effectors, the cyclomodulins, that subvert normal host-cellular processes by inducing cell-cycle arrest (Taieb et al., 2011). The eukaryotic cell cycle consists of the gap G1 phase characterized by cell growth, the S phase characterized by DNA replication, the second gap G2 phase in which cells are prepared for division, the M phase during which mitosis take place, and the G0 phase when cells can enter a quiescent state. The cell cycle progression is regulated by complexes consisting of cyclins and enzymes, the cyclin-dependent kinases (CDK) (Bertoli et al., 2013). Cell-cycle phase related accumulation or degradation of cyclins regulates their association with CDKs and subsequently controls cell cycle progression and cell division. Each of the cyclin-CDK complexes modifies specific sets of proteins by phosphorylation to guarantee a chronological cell cycle progression (Lim and Kaldis, 2013).

Until recently only few studies have addressed the influence of *Staphylococcus aureus* on host cells proliferation and differentiation. *S. aureus* epidermal cell differentiation inhibitor (EDIN) affects the differentiation of cultured keratinocytes (Sugai et al., 1992). Exposure of keratinocytes to staphylococcal alpha-toxin almost doubled the interval of the S+G2/M phase (Haugwitz et al., 2006). Beside these two compounds there are also other *S. aureus* toxins that interfere with host cell cycle. The phenol-soluble modulins (PSM) delay the cell cycle in the G2 phase (Deplanche et al., 2015), and the staphylococcal enterotoxin O toxins (SEIO) delay the G1 phase (Hodille et al., 2016). The *S. aureus*-induced delay in the eukaryotic cell cycle is normally associated with an increased bacterial infectivity and compromised immune system, suggesting, that the capacity of *S. aureus* to alter the host cell cycle contributes to virulence and host cell invasiveness (Alekseeva et al., 2013).

Whether PSMs and SEIO are the only staphylococcal compounds that caused host cell cycle arrest is questionable. Recently it has been shown that the “*l*ipo*p*rotein-*l*ike” genes (*lpl*) encoded on the νSaα islands (Baba et al., 2008) in *S. aureus* contribute to internalization into non-professional antigen presenting cells such as keratinocytes (Nguyen et al., 2015). They also cause an enhanced invasion into murine skin and an increased bacterial burden in a murine kidney abscess, suggesting that the *lpl* gene cluster serves as an important virulence factor (Nguyen et al., 2015).

Here we investigated whether Lpl proteins have an effect on the progression of eukaryotic cells. We show that *S. aureus* USA300 induces a significant G2/M phase transition delay in HeLa cells compared to the *lpl* deletion mutant. Comparative analysis of lipidated and unlipidated Lpl1, a representative of Lpls, shows that the protein part delays G2/M phase transition in HeLa cells. Taken together, the Lpl proteins encoded on the νSaα island significantly interferes with the cell cycle.

## Materials and methods

### *Staphylococcus aureus* strains and culture conditions

*Staphylococcus aureus* USA300, its *lpl* deletion mutant USA300Δ*lpl* and the complemented mutant USA300Δ*lpl* (pTX30-*lpl*) are constructed as previously described (Nguyen et al., 2015). All *S. aureus* cultures were performed as follows: Staphylococci were grown overnight in Brain Heart Infusion (BHI) broth, aliquots were diluted (1:50) in DMEM and cultivated at 37°C under anaerobic conditions until an OD of 0.6 at 600 nm was reached, corresponding to 10^8^ CFU/ml (CFU, colony-forming unit). Bacteria were harvested by centrifugation, washed twice with phosphate-buffered saline (PBS), and suspended in the interaction medium (DMEM). Bacterial concentrations were estimated spectrophotometrically and confirmed by plate counts.

### Expression and purification of Lpl1(+LSP) and Lpl1(−LSP)

The purification was carried out essentially as described previously (Nguyen et al., 2015). Lpl1(+LSP) was isolated from the membrane fraction of *S. aureus* SA113 (pTX30::*lpl1*-his) whereas Lpl(−LSP) was isolated from the cytoplasmic fraction of *S. aureus* SA113Δ*lgt* (pTX30::*lpl*1(-sp)-his). Both of these Lpl1-his proteins were purified via Ni-NTA affinity chromatography. For the enhance of protein expression, the clones were first cultivated aerobically at 37°C in the absence of xylose (BO-medium) until OD_578 nm ≈_ 0.5 was reached, then following continuously cultivated for 4 h in the presence of 0.5% xylose to induce the Lpl1 expression. The bacterial cells were harvested and washed two times with Tris buffer (20 mM Tris, 100 mM HCl, pH 8.0). Then the pellet was re-suspended with Tris buffer containing protease inhibitor table (Merck, Darmstadt, Germany) and lysostaphin (30 μg/ml) and incubated at 37°C for 2 h to disrupt the cell wall. After the first ultracentrifugation (235,000 × g for 45 min at 4°C), the supernatant containing the cytoplasmic proteins were collected for the next purification step. For membrane fraction isolation, the pellet was subsequently dissolved overnight at 6°C with Tris buffer containing 2% Triton-X100. After the second ultracentrifugation step, the supernatant containing membrane fragments was collected. The purification step was carried with Ni-NTA supper flow beads (Qiagen, Germany). The Ni-NTA beads capturing Lpl1 proteins were washed four times with the first washing buffer (Tris buffer containing 0.25% TritonX-100 and 20 mM imidazole) and following 2 times with the second washing buffer (Tris buffer containing 0.25% TritonX-100 and 40 mM imidazole). Finally, the Lpl1 was eluted with the same buffer containing 500 mM imidazole. The Lpl1 were concentrated via centrifugal ultra-filter unit with a molecular mass cut-off of 10 kDa (Sartorius AG, Göttingen, Germany). The concentrated samples of Lpl1 were dialyzed overnight at 6°C with DPBS buffer (Life technologies, Darmstadt) by Dialyzer tube, MWCO 6–8 kDa (Merk, Darmstadt) and was subsequently lyophilized overnight. The lyophilized samples stored at 4°C for further experiment. Two microgram of lyophilized samples were dissolved in water and run for SDS page to check the quantity and qualification of purified protein samples.

### Maintenance of eukaryotic cells

The human cervix cancer HeLa cells were obtained from American Type Culture Collection, Manassas, VA, USA HeLa cells were cultured in DMEM (Dulbecco's modified Eagle medium, Gibco, Invitrogen Corporation, Cergy Pontoise, France) supplemented with 10% fetal calf serum (FCS) (BioWest, Paris, France), 100 U/ml penicillin and 100 μg/ml streptomycin. Trypsin/EDTA (Gibco, Saint Aubin, France) was used to release adherent cells for subculturing.

### Cell synchronization: double thymidine block (DTB)

Cell cycle analysis experiments were performed by a standard method with synchronized cells when at a given time point the majority of cells are in a particular cell cycle phase. It allows us to detect even little variations in cell cycle phase distribution. In order to synchronize the cells at the G1/S border the double thymidine block (DTB) protocol was employed as described (Deplanche et al., 2015). Briefly, HeLa cells were grown in a 25-ml flask up to 30% confluence. After washing with PBS, cells were cultivated in DMEM containing 10% of FCS supplemented with 2 mM thymidine (DMEM-T) for 18 h. Thymidine was then removed by washing with PBS and cells were cultivated in fresh DMEM with 10% of FCS for 9 h to release cells. The cells were then cultivated in DMEM-T for 17 h, followed by cultivation in DMEM containing 10% of FCS.

### Exposition of HeLa cells to *S. aureus* strains or to purified Lpl1

HeLa cells were grown in 25 ml flasks (30% confluence at the beginning of DTB), then cells were infected with *S. aureus* at a multiplicity of infections (MOI, number of bacteria per cell at the onset of infection) of 60:1, for 16, 20, 25, and 28 h after DTB release (infection medium: DMEM). The incubation time was chosen in agreement with the recognized evaluation of the phases of HeLa cell cycle and on the basis of our preliminary experiments. There was no alteration between the phases of infected and uninfected cells before 25 h of incubation of HeLa cells with bacteria. Consequently, synchronized HeLa cells were exposed to bacteria for 20, 25, and 28 h after DTB release. HeLa cell concentrations were determined using one of the four samples. The remaining samples were used for the analysis in triplicate. The low HeLa cell density at the beginning of the experiment was used to ensure cell proliferation during the entire experiment since cells cease proliferate, when they reach confluence (Owen et al., 1989).

Bacterial concentrations were estimated spectrophotometrically and were confirmed by CFU determination. Unbound bacteria were removed 2 h post-infection by treating cells with 20 μg/ml of lysostaphin and 100 μg/ml gentamicin for 2 h in cDMEM containing 3% FCS to eliminate the extracellular bacteria (Schmaler et al., 2009). Then infected HeLa cells were cultivated in cDMEM containing 25 μg/ml of gentamicin.

In experiments assigned for the analysis of the impact of purified Lpl1-his on the cell cycle progression, HeLa cells were exposed to 2 and 5 μg/ml of Lp1 and Lpl1(−LSP) for 20, 25, and 28 h after DTB release; quite similar as described for the exposure to bacteria. We also used 2 and 5 μg/ml of Pam2C (Pam_2_CSK_4_) and Pam3C (Pam_3_CSK_4_) as controls. Pam2C and Pam3C are synthetic di- and tri-palmitoylated lipopeptides (obtained from EMC, Tübingen, Germany) mimic the acylated amino terminus of bacterial lipoproteins. In all cell cycle progression experiments with either whole bacteria or purified proteins there was no difference before 20 h exposure, however, there was strong effect after 25 h and only weak effect after 28 h exposure. Consequently, we presented the values after 25 h exposition. It is worth mentioning that the recognized duration of phases of the HeLa cell cycle is approximately 10, 8, 3, and 1 h, for G1, S, G2, and M, respectively (Comayras et al., 1997).

### Cell cycle determination by flow cytometry analysis

After 25 h post-infection cells were detached and combined with adherent cells, which were collected by Trypsin/EDTA treatment and fixed in 70% ethanol overnight. Cells were then stained with propidium iodide (PI) and analyzed with an Accuri C6 flow cytometer (Becton Dickinson, Le Pont de Claix, France) as described earlier (Alekseeva et al., 2013; Deplanche et al., 2015). Data were collected from 20,000 cells and analysis was performed with CFlow software.

### Invasion assays

Invasion assays was performed according Almeida et al. with slight modifications (Almeida et al., 1996). Briefly, 3 × 10^6^ HeLa cells were incubated in cDMEM in 12 well plates overnight at 37°C in 5% CO_2_. Then cells were exposed to *S. aureus* strains: USA300, its deletion mutant USA300Δ*lpl* and the complemented mutant at MOI 100:1 (with inoculum 2 × 10^7^ CFU/well) for 1 h. Afterwards, cells were washed and incubated in DMEM containing 20 μg/ml lysostaphin and 100 μg/ml gentamicin for 2 h in order to remove extracellular bacteria and to measure internalized intracellular bacteria only. After lysis with 0.05% Triton X-100 in PBS, cell lysates were plated on BHI agar, and CFU were determined after overnight incubation using a micromethod (Florence Baron et al., 2006). To compare the invasion rates of USA300 and its mutants the percentage of internalized bacteria from the inoculum (i.e., of the number of input CFU) was determined for each strain.

### LDH cytotoxicity test

Different concentration of purified Lpl1(−LSP) were tested for cytotoxic activity against HaCaT cells, an immortalized human keratinocyte line by using the LDH cytotoxicity assay kit (Thermo Scientific). The experiments were performed in triplicate in a 96 well plates. First, the optimal human cell number was determined to ensure LDH signal is within the linear range. Then we selected the initial seeding number of 10^4^ cells in 100 μl media (DMEM high glucose, 10% FBS, 1% Pen/Strep) per well (4 × 10^4^/100 μl/a well). After 2 days incubation at 37°C with 5% CO_2_, the HaCaT cells were incubated with 10 μl purified Lpl1(−LSP) of different concentrations for 1 and 25 h, respectively. We also used PBS buffer and PBS buffer with 0.25% of Trixton X-100 as controls. After incubation cells were centrifuged and the LDH activity determined in the supernatant by measuring the absorbance at 490 nm and 680 nm in a Tecan reader. The percent cytotoxicity was calculated following the instruction of the kit.

### Hemolysis activity assay

*Staphylococcus aureus* USA300 and *lpl* mutant strains were aerobically cultured in TSB medium for 12 h and subsequently cultured on sheep blood agar at 37°C for 18 h. After that, these plates were stored at 4°C for 12 h for a cold-shock reaction. Beta toxin is known as a hot-cold toxin, which interacts with sheep blood cells at 37°C and only lyses them at 4°C (Herbert et al., 2010). The hemolytic activity makes the clear zone on the agar around the *S. aureus* colonies.

### HPLC analysis of PSM peptides

The strains *S.a* USA 300 wild type, USA 300Δ*lpl*, and the complemented USA 300 Δ*lpl* (pTX30-*lpl*) were grown overnight in TSB at 37°C and centrifuged for 10 min at 5000 × g at 4°C. The corresponding supernatants were filtered through sterile syringe filter with 0.2 μm pore size (Sarstedt, Germany) prior to be concentrated 5x using speedvac vacuum concentrator. PSM peptides were separated from the supernatant by reversed-phase chromatography using an XBridge C8 5 μm, 4.6 × 150 mm column (Waters). A linear gradient from 0.1% TFA in water to acetonitrile containing 0.08% TFA for 15 min with additional 5 min of 100% B at a flow rate of 1 ml/min was used and a 50 μl sample volume was injected. Peaks were detected at 210 nm. The PSM peptides were eluted between 12 and 19 min. As standard PSMα, PSMβ, and delta-toxins.

### Statistical analysis

At least three different assays were performed per experiment. The differences among the groups were assessed by analysis of variance (ANOVA). *P* < 0.05 were considered to be significant. Tukey's Honestly Significant Difference test was applied for comparison of means between groups. The values are expressed as mean ± standard deviation (±SD).

## Results

### The *S. aureus* lpl cluster Is responsible for the host cell cycle alteration

After double thymidine block (DBT) HeLa cells were synchronized at the border of G1/S phase in agreement with the evaluated phases of the HeLa cell cycle. Cell cycle experiments were carried out with *S. aureus* USA300 carrying the νSaα island, its Δ*lpl* deletion mutant and the complemented mutant; the genetic organization is shown in Figures S1A,B. Exposure of HeLa cells to USA300 or its Δ*lpl* deletion mutant for 20 h showed hardly a difference in cell cycle. However, after 25 h there was a strong difference between wild type and the mutant, which was less pronounced after 28 h. Therefore, cell cycle analysis was carried out after 25 h of cells exposed to *S. aureus*. The rather narrow time frame of cell cycle arrest can be explained by the onset of cellular repair systems as has been shown for example by DNA-damage (Siegl and Rudel, 2015); however, such stress situations can also trigger the destruction of the damaged cell and activation of pathways leading to programmed cell death if cellular damage cannot be precisely repaired (Nascimento et al., 2012).

**Figure 1:**
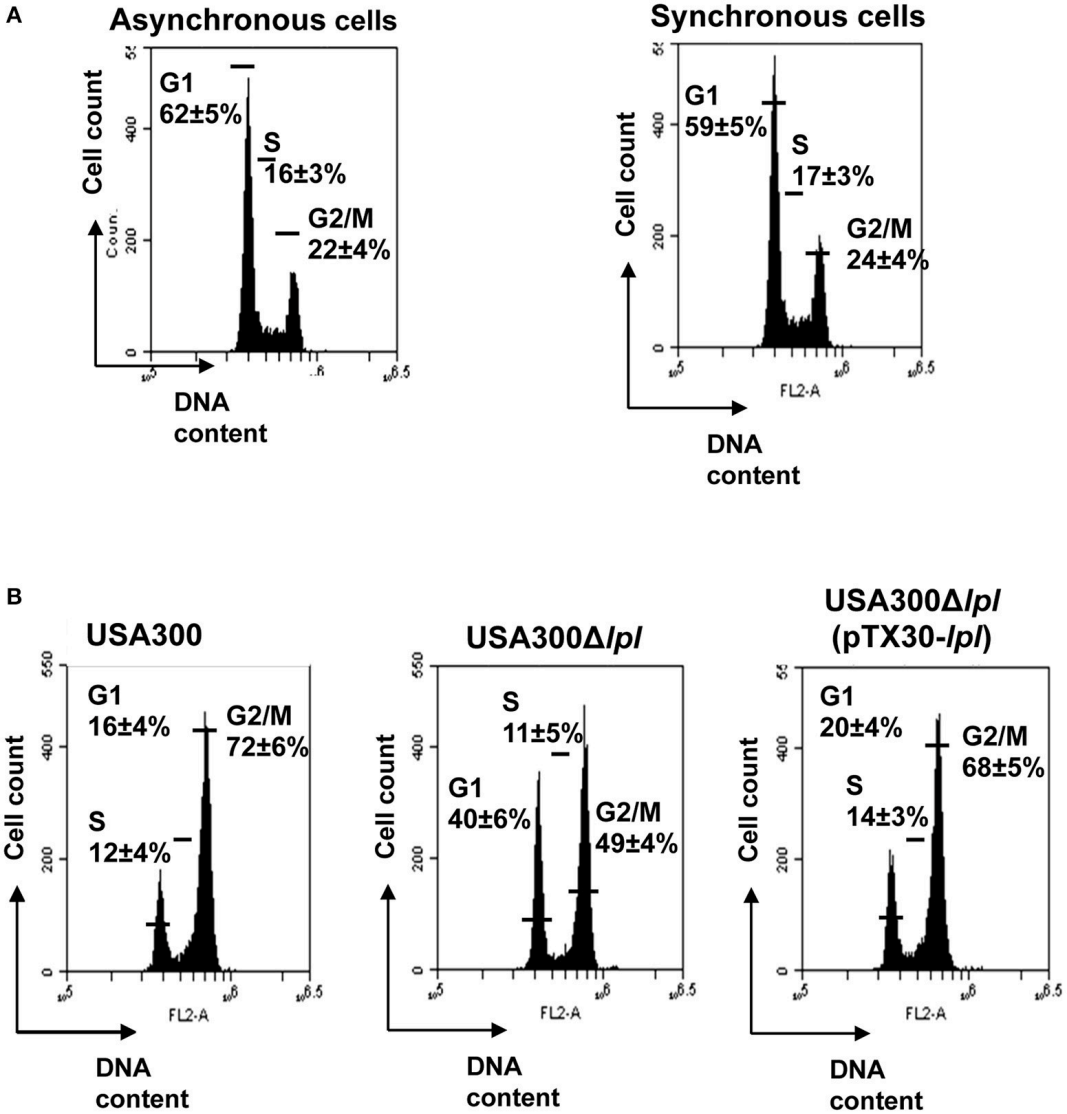
**Effect of whole *S. aureus* cells on HeLa cell cycle. (A)** Comparison of cell cycle phases of asynchronized and synchronized HeLa cells after 25 h incubation (control experiment) reveal a comparable distribution (%) of cells in G1 (±60%), S (±17%), and G2/M (±23%) phases. **(B)** Effect of whole *S. aureus* cells on cell cycle progression: *S*. *aureus* USA300, USA300Δ*lpl* and complemented mutant USA300Δ*lpl* (pTX30-*lpl*). Twenty-five hours post-infection, cells were detached and together with adherent cells they were fixed in 70% ethanol overnight, stained with PI and analyzed by flow cytometry. Data were collected from 20,000 cells and analysis was performed with CFlow Accury6 software. The average percentage of cell cycle phase ± SD is indicated. The values of one representative experiment, out of four, is shown. *P*-values < 0.05 were considered to be significant.

The DNA content of untreated synchronized HeLa cells 25 h after DBT release was used as a control: 59 ± 5% of cells were in the G1, 17 ± 3% in the S and 24 ± 4% in the G2 phase (Figure 1A). Exposure of HeLa cells to *S. aureus* USA300 caused a significant delay in the G2/M phase: after 25 h post-infection only 16 ± 4% of cells were in G1, 12 ± 4% in S, and 72 ± 6% were still in the G2/M phase (Figure 1B). When HeLa cells were infected with the *lpl* deletion mutant, USA300Δ*lpl*, only 49 ± 4% were in the G2/M phase; while with the complemented mutant, USA300Δ*lpl* (pTX30-*lpl*), 68 ± 5% of cells were in the in G2/M phase indicating that almost wild type levels were regained (Figure 1B). The results demonstrate that in the presence of USA300 and the complemented *lpl* mutant the G2/M phase transition was significantly delayed compared to the *lpl* deletion mutant, indicating that the *lpl* gene cluster is responsible for this delay. The next question was whether a purified Lpl protein is also able to delay the G2/M phase transition.

### Comparative analysis of the effect of lipidated and unlipidated Lpl1 on HeLa cell cycle delay

To verify whether protein or lipid portion of lipoprotein is responsible for a G2/M phase transition delay, we selected Lpl1, as a prototype of the cluster, which is encoded by the first gene of the *lpl* cluster in USA300 (Figure S1A). Lpl1 was expressed with C-terminal His-tag in SA113 and SA113Δ*lgt* with and without lipoprotein signal peptide (LSP). In the absence of LSP Lpl1 was not expressed in the membrane but in the cytoplasm. The plasmid encoded constructs for expression of Lpl1, carrying the lipid modification, and Lpl1(−LSP), not lipid modified, are shown in Figures S1C,D. The lipid modified Lpl1 was expressed in SA113 (pTX30::*lpl1*-his) with its LSP; Lpl1(+LSP) was isolated from the membrane fraction and purified by Ni-NTA chromatography and its purity verified by SDS-PAGE (Figure S1E). In *S. aureus* the lipid moiety of lipoproteins is tri-acylated (Kurokawa et al., 2009). As we also wanted to see whether the protein part alone, has an effect on the cell cycle, we expressed Lpl1 without its lipoprotein signal peptide in SA113Δ*lgt* (pTX30::*lpl1*-(LSP)-his). This Lpl1(−LSP) was isolated from the cytoplasm and also purified by Ni-NTA chromatography (Figures S1D,E).

**Figure 2:**
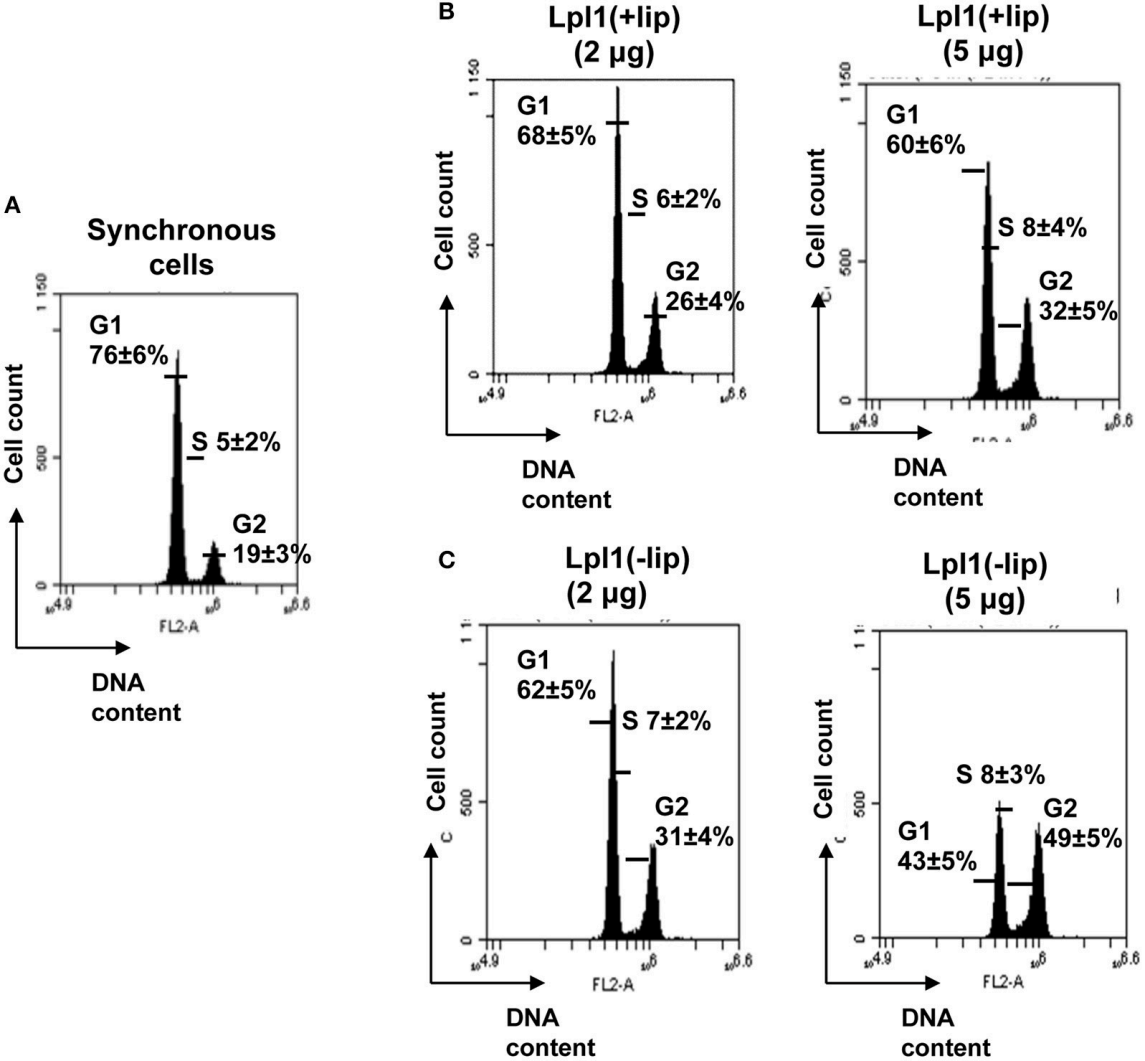
**Effect of Lpl1(+LSP) and Lpl1(−LSP) on HeLa cell cycle. (A)** Control experiment: In synchronized HeLa cells after 25 h incubation the distribution (%) of cells in G1 phase was (±76%), in S (±5%), and in G2 (±19%). Effect of 2 and 5 μg/ml of purified **(B)** Lpl1(+LSP) (68 and 171 nM) and **(C)** Lpl1(−LSP) (70 and 176 nM) on cell cycle progression. The average percentage of cell cycle phase ±SD is indicated. We show the values of one representative experiment out of four. *P*-values < 0.05 were considered to be significant.

Analysis of the DNA content of control untreated synchronized HeLa cells 25 h after DBT release revealed that 76 ± 6% of cells were in the G1, 5 ± 2% in the S and 19 ± 3% in the G2/M phase (Figure 2A). In the presence of 2 and 5 μg/ml (68 and 171 nM) Lpl1 the percentage of HeLa cells after 25 h post incubation was increased from 19 ± 5% (control) to 26 ± 4% and 32 ± 5% in G2/M phase, respectively (Figure 2B). This result shows that Lpl1(+LSP) moderately delayed the G2/M transition phase. Next we analyzed Lpl1(−LSP) in the same way as Lpl1(+LSP) and it turned out that with Lpl1(−LSP) (70 and 176 nM) even a higher percentage of the HeLa cells (31 ± 4 and 49 ± 5%) were in the G2 phase after 25 h (Figure 2C). This result demonstrates that solely the protein portion of Lpl1 causes the G2/M transition delay.

### The synthetic lipopetides Pam2C and Pam3C also extended the G2 phase

**Figure 3:**
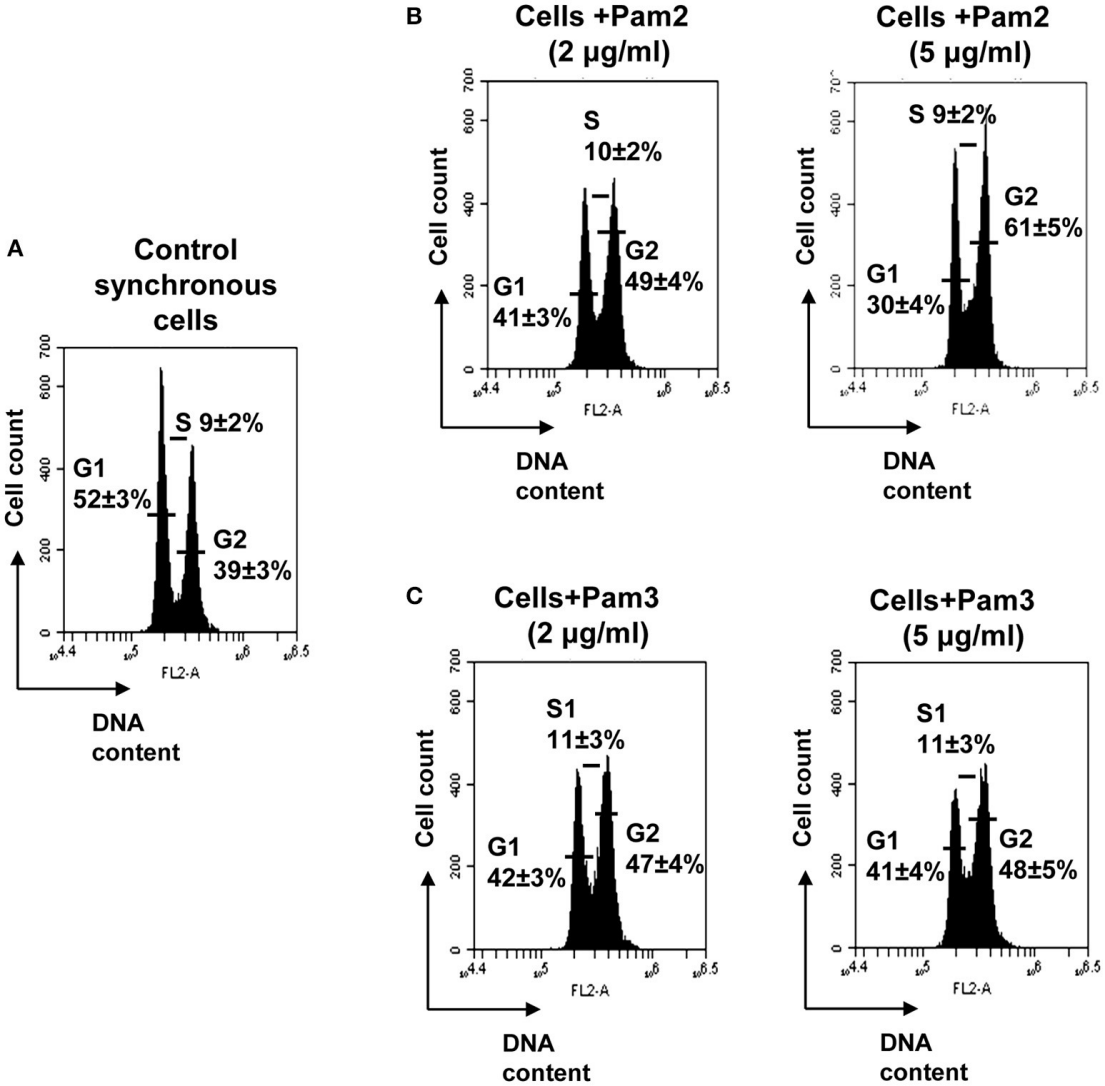
**Effect of the synthetic lipopeptides Pam2 and Pam3 on HeLa cell cycle. (A)** Control experiment: In synchronized HeLa cells after 25 h incubation the distribution (%) of cells in G1 phase was (±52%), in S (±9%), and in G2 (±39%). Effect of 2 and 5 μg/ml of **(B)** Pam2 (1574 and 3937 nM) and **(C)** Pam3 (1333 and 3333 nM) on cell cycle progression. The average percentage of cell cycle phase ± SD is indicated. The values of one representative experiment, out of four, is shown. *P*-values < 0.05 were considered to be significant.

To confirm our observation of a pivotal role of the protein portion of Lpl1 in the inhibition of the G2/M phase transition, we examined whether synthetic lipopeptides have an impact on cell cycle alteration. Therefore, synchronized HeLa cells were exposed to 2 and 5 μg/ml of Pam2 and Pam3. These are synthetic lipopeptides that mimic the acylated amino terminus of bacterial lipoproteins; the structure is shown in Table 1. Analysis of the DNA content of untreated synchronized HeLa cells 25 h after DBT release revealed that 52 ± 3% of cells were in the G1 phase, 9 ± 2% in the S phase and 39 ± 3% in the G2 phase (Figure 3A). Exposure of HeLa cells to 2 and 5 μg/ml (1574 and 3937 nM) of Pam2 resulted in an increase of cells in the G2 phase to 49 ± 4 and 61 ± 5%, respectively (Figure 3B). Interestingly, exposure HeLa cells to 2 and 5 μg/ml (1333 and 3333 nM) Pam3C showed less influence on the cell cycle. The percentage of cells in the G2 phase was increased from 39% (control) to 47% (2 μg/ml Pam3C) and to 48% (5 μg/ml Pam3C), respectively. Unlike Pam2Cys there was no dose dependent increase with Pam3C (Figure 3C). As we used a more than 20 times higher concentration of Pam2C and Pam3C compared to Lpl1 to see an effect at al, we assume that the lipopeptides have only a marginal effect on cell cycle. These results strengthen our findings that the protein portion of Lpl1 is the major player in the eukaryotic cell cycle alteration.

**Table 1:** **The list of compounds used in this study**.

**Compounds**	**Structures**	**Molecular weight (g/mol)**
Lpl1(+LSP)	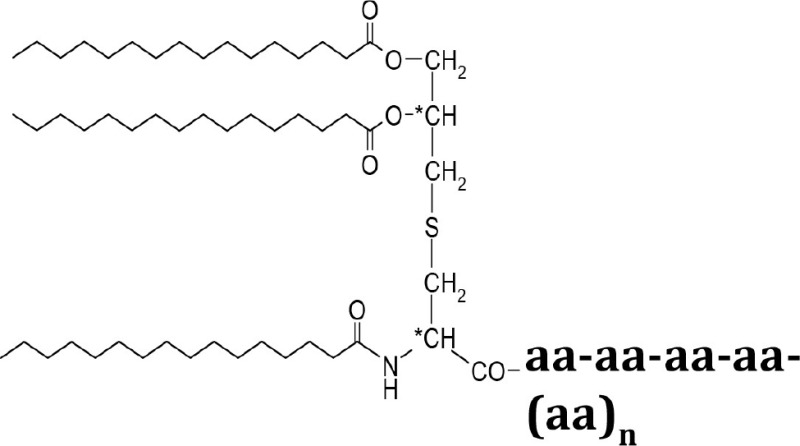	29210
Lpl1(−LSP)	**aa-aa-aa-aa-(aa)_n_**	28350
Pam3Cys (Pam3CSK_4_)	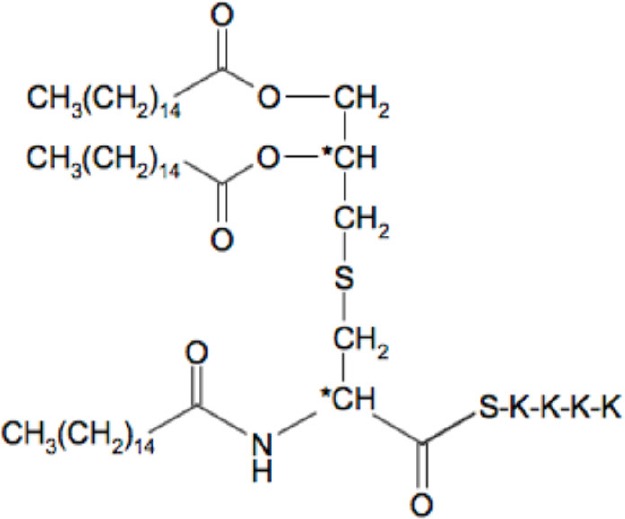	1509
Pam2Cys (Pam3CSK_4_)	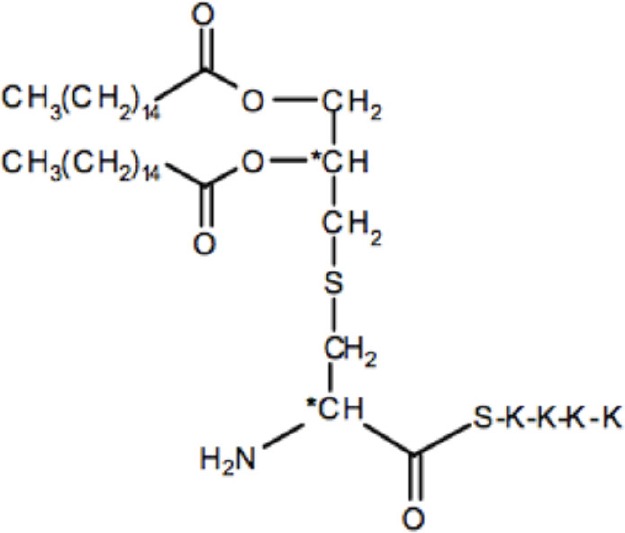	1271

### The lpl cluster contributes to *S. aureus* internalization into HeLa cells

**Figure 4:**
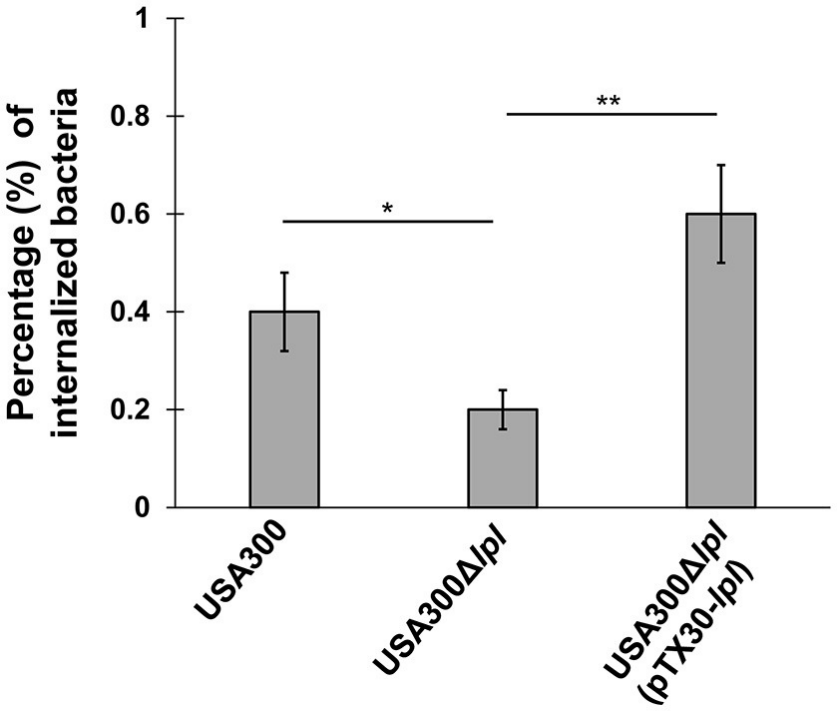
**Invasion assay of *S. aureus* in HeLa cells**. HeLa cells (3 × 10^6^) were incubated in cDMEM overnight. Then cells were exposed to USA300, USA300Δ*lpl* and the complemented mutant USA300Δ*lpl* (pTX30-*lpl*) at a MOI 100:1 (with inoculum 2 × 10^7^ CFU/well) for 1 h. Afterwards, cells were incubated with 20 μg/ml of lysostaphin and 100 μg/ml gentamicin for 2 h in order to remove extracellular bacteria. After lysis with 0.05% Triton X-100, CFU of cell lysates were determined by a micromethod. Three independent experiments were performed in triplicates.The data were presented as percentage of internalized bacteria (CFU of internalized bacteria divided by CFU of bacteria added to cells, multiplied by 100). ^*^P-values < 0.05, ^**^P-values < 0.01 and *P*-values < 0.05 were considered to be significant.

It has been indicated that in the presence of the *lpl* cluster enhances the invasion into human keratinocytes and in mouse skin (Nguyen et al., 2015). As we study here the effect on cell cycle in HeLa cells it is important to verify whether the same results were achieved in this cell line. Indeed, the internalization into epithelial cells was lowest with USA300Δ*lpl* and was increased two-fold and even three-fold with USA300 (parent) and the complemented mutant USA300Δ*lpl* (pTX30-*lpl*), respectively (Figure 4). These results clearly demonstrate that the lpl cluster contributes to HeLa cell invasion and that there might be a link between host cell invasion and G2/M cell cycle delay.

### The presence or absence of the lpl cluster has no effect on hemolysis activity or PSM production

**Figure 5:**
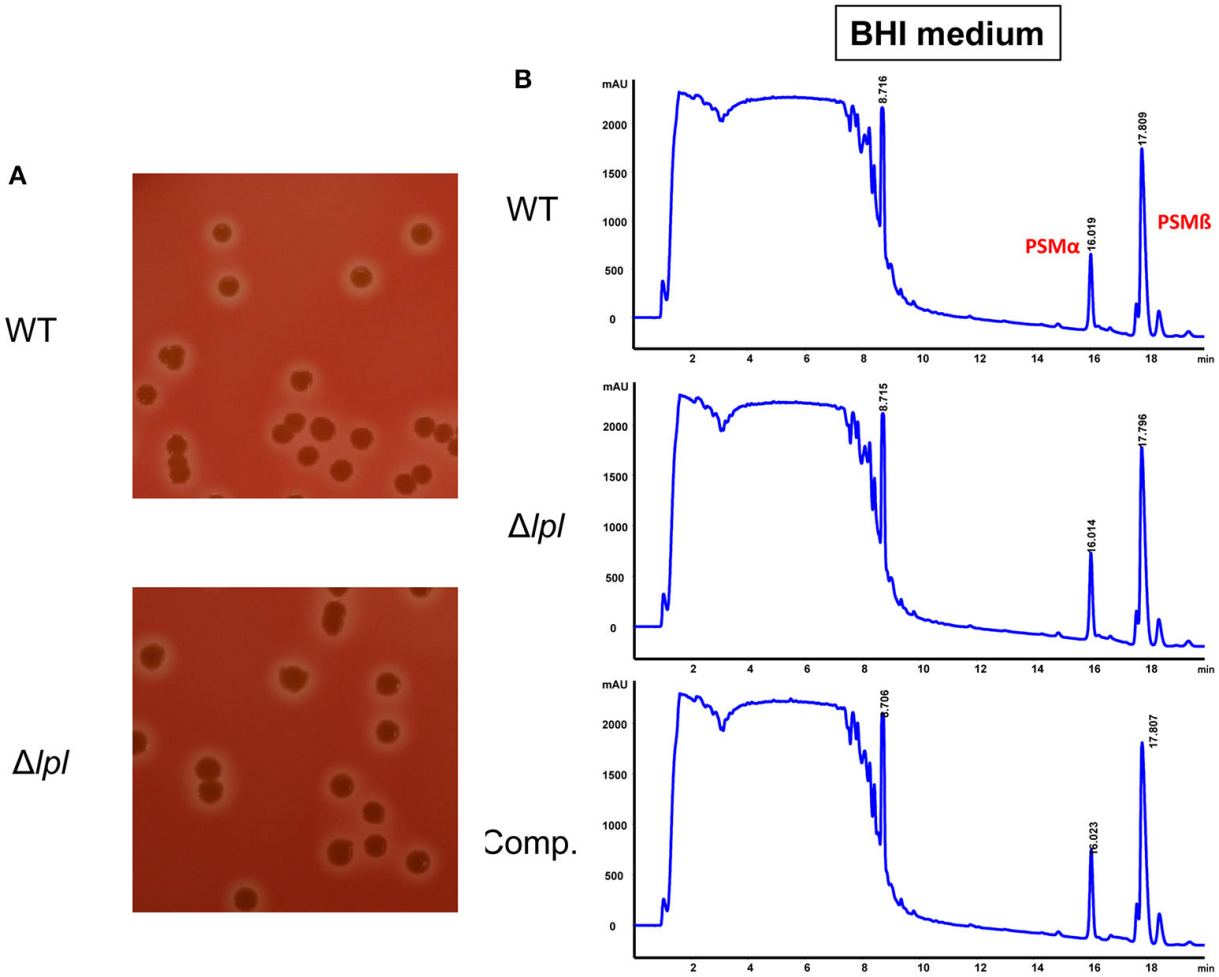
**Comparative alpha-hemolysis activity and PSM production in USA300 and the Δ*lpl* mutant. (A)** Hemolysin activity on sheep blood agar. *S. aureus* strains were grow on sheep blood agar at 37°C for 18 h, following a cold shock at 4°C for 12 h. The beta toxin lysed the erythrocytes of sheep blood cells and created a clear halo. **(B)** HPLC analysis of PSMs in the supernatants of USA300, Δ*lpl* mutant, and the pTX30-*lpl* complemented mutant. Cultures were grown aerobically overnight in BHI medium at 37°C.

It has been shown that alpha-hemolysin and PSMs have an impact on cell cycle alteration (Haugwitz et al., 2006; Deplanche et al., 2015). In order to rule out that altered expression of these compounds have an influence on cell cycle delay we investigated whether in USA300Δ*lpl* alpha-hemolysin and PSM was altered compared to the parent strain USA300. We found that USA300 and USA300Δ*lpl* showed no difference in any of the two parameters. Alpha-hemolysin production, as indicated by the hemolysis zone on sheep agar plates, was unaltered in the mutant (Figure 5A). The contents of PSM-α, -β, and -γ in the supernatant of USA300 and its *lpl* mutant were determined by HPLC analysis; there was no difference in the production of the PSM peptides (Figure 5B). As the *S. aureus* strains were pre-cultivated in BHI medium for cell cycle experiments the quantification of the PSMs was also carried out in supernatant of BHI grown cells. Interestingly, when *S. aureus* cells were cultivated in TSB medium the HPLC resolution of PSMs was improved, but there was also no difference in the concentration between wild type and Δ*lpl* mutant (Figure S2). These results indicate, that the observed effect of whole *S. aureus* cells as well of the isolated Lpl1(+LSP) and Lpl1(−LSP) on extending the G2/M transition phase are really due the lpl cluster and the Lpl proteins.

## Discussion

The damage of an epithelium by a bacterial infection is counteracted by constant shedding a renewal of epithelial cells (Vielfort et al., 2013). Many bacteria can interfere with the cell cycle progression thus impairing the integrity of epithelial barriers and allowing pathogens to successfully colonize and to invade host cells (Nougayrède et al., 2006). To avoid extensive damage, the various phases of cell proliferation are controlled by checkpoint pathways to ensure a successful completion of the preceding cell cycle phase (Murray, 1993; Rhind and Russell, 2012). CDKs plays a crucial role at the checkpoint. They may slow down the cell progression or induce an arrest of a cell division in response to dangerous external stimuli (Alekseeva et al., 2013; Duronio and Xiong, 2013).

Many bacterial pathogens were shown to interfere with the host cell cycle. For example cyclomodulins are bacterial toxins that can inhibit or stimulate the eukaryotic cell cycle thus playing a role in infection (Nougayrede et al., 2005; Oswald et al., 2005). *Escherichia coli* for example produces a variety of effectors: the cycle inhibiting factors (Cifs), Cytolethal Distending Toxins CDTs), Shiga toxin (Stx), Cytotoxic Necrotizing Factor 1 (CNF1), or the hydrolase γ-glutamyltranspeptidase (γ-GT); they all delay cell cycle progression in different phases (Nougayrède et al., 2001; Nougayrede et al., 2005). Cif, a G_2_-checkpoint inducer is in so far a unique cyclomodulin as its activity is independent of the DNA-damage pathway but it usurps the ubiquitin-dependent degradation pathway of host cells (Taieb et al., 2011). While *Shigella* and pathogenic *E. coli* block host cells in the G2/M phase transition (Nougayrède et al., 2006; Taieb et al., 2006; Iwai et al., 2007), *Neisseria gonorrhoeae* and *Porphyromonas gingivalis* inhibit cell proliferation via G1 arrest (Jones et al., 2007; Inaba et al., 2009).

Studying the cytopathic effect of *S. aureus* on human HeLa and bovine MAC-T epithelial cell lines indicated that invasion of *S. aureus* into these host cells not only slowed down their proliferation but also caused enlargement of the cells and delayed the G2/M phase transition (Alekseeva et al., 2013). Not in the G1 phase, but in the G2 phase-enriched cells the number of internalized *S. aureus* cells was significantly increased suggesting that in the G2 phase staphylococcal internalization and/or intracellular proliferation was enhanced. A similar effect was observed with *Toxoplasma gondii* that exploits UHRF1 and induces host cell cycle arrest at G2 to enable its proliferation (Brunet et al., 2008).

As all the effects of *S. aureus* were only seen with live but not with heat-killed bacteria it was suggested that cell wall structures such as peptidoglycan, lipoteichoic acid or wall teichoic acid are not involved in cell cycle modulation but rather secreted compounds (Alekseeva et al., 2013). Indeed, it has been shown that the so far described cyclomodulins of *S. aureus* represent secreted toxins such as α-toxin (Hla) that increases the continuation of S+G2/M phases (Zavizion et al., 1995; Haugwitz et al., 2006), epidermal cell differentiation inhibitor toxin (EDIN) that suppresses keratinocyte differentiation (Sugai et al., 1992; Munro et al., 2010), phenol-soluble modulin alpha peptides (PSMα) that induces G2/M transition delay (Deplanche et al., 2015), and staphylococcal Enterotoxin O (SEIO) that caused G0/G1 phase delay (Hodille et al., 2016). Since Hla and PSMα destroy the host membrane and cause cell lysis of many human cell types (Berube and Bubeck Wardenburg, 2013; Cheung et al., 2014), it is not astonishing that they also affect cell growth. More surprising is that they specifically delay the cell cycle progression in the G2 phase (Deplanche et al., 2015), a comparatively short interphase characterized by rapid growth, development of chromatin and nucleus and the preparation for mitosis.

The G2/M phase transition delay by Hla and PSMα could be due to inhibition of the dephosphorylation of Cdk1 and phosphorylation of histone H3 observed with whole *S. aureus* cells (Alekseeva et al., 2013). The cyclin-dependent kinase Cdk1 is one of the key players of the G2-M transition and its dephosphorylation at the end of the G2 phase is essential for triggering mitotic entry (Lindqvist et al., 2009). Phosphorylation of histone H3 at serine 10 starts in the prophase of mitosis and is essential for chromosome condensation and segregation *in vivo* (Wei et al., 1999). Inhibition of dephosphorylation of Cdk1 and phosphorylation of histone H3 is most likely the reason why infected cells failed to enter mitosis and accumulated in the G2 phase. SEIO's potential partner could be cullin-3 that is involved in the transition from G1 to S phase (Hodille et al., 2016).

When we found that the tandem lipoprotein cluster of the νSaα, genomic island in *S. aureus* contributes to host cell invasion (Nguyen et al., 2015), we wondered whether the lpl cluster has also an impact on host cell cycle. Indeed, we show here that in the presence of the lpl cluster the G2/M phase transition is markedly delayed in HeLa cells and that the lpl cluster also caused an increased invasiveness in this cell line. Whether both activities are correlated we don't know. However, it could be that the extension of the G2 phase conditions increased invasion frequency. As it has been shown previously that Hla and PSMα also cause G2/M transition delay we confirmed that there was no difference in hemolysis activity and PSM production in USA300 and its Δ*lpl* mutant (Figure 5).

The *lpl* gene cluster encodes nine highly homologous lipoproteins (Nguyen et al., 2015; Shahmirzadi et al., 2016). As all nine lipoproteins were difficult to analyze and as antibodies against one cross-reacted with the others, we have chosen one lipoprotein, Lpl1 as a representative to answer the question whether it causes a G2/M phase transition delay. Indeed, purified Lpl1 caused a G2/M phase transition delay. While lipid modification of the Lpls' is absolutely necessary for TLR2-meditated immune stimulation, it is not necessary for the extension of the G2/M transition delay. This makes sense as the protein portion is exposed at the bacterial cell surface and makes contact with a so far unknown host cell compound thus triggering the G2/M transition delay. We assume that the Lpls' mediated G2/M transition delay is the mechanistic basis for the observed increased host cell invasion. At least it has been shown that bacterial invasion occurs mainly in the G2 phase (Alekseeva et al., 2013).

Since Lpl1 shares high sequence homology with the other eight Lpl proteins (Lpl2-9) (Figure S1A) we assume that they also have a similar activity. Anyway, the *S. aureus* lpl lipoproteins represent a specific class of lipoproteins most abundant in pathogenic epidemic strains. There is so far no indication that they are involved in substrate transport like many other lipoproteins in *S. aureus* (Shahmirzadi et al., 2016). Like most other lipoproteins they contribute to TLR2-dependent immune signaling for which the lipid modification is essential; without the lipid part Lpl1 is completely immune silent (Nguyen and Götz, 2016). Therefore, we assume that the lpl cluster has essentially a role in pathogenicity such as immune evasion by increasing host cell invasion (Nguyen et al., 2015). Once inside the host cell bacteria are largely protected from the immune response and also from antibiotic attack. Another interesting feature of the *lpl* gene cluster are the tandemly organized genes. Most likely they are created by multiple recombination events at the central conserved region and by shuffling two variable regions in one gene thus maintaining a conserved unit (Tsuru and Kobayashi, 2008).

The *S. aureus* lpl cluster is reminiscent of the lipoproteins in the Gram-negative spirochetes such as *Treponema pallidum* and *Borrelia burgdorferi*, in which lipoproteins have an immunomodulatory effect comprising both immune evasion and immune activation (Kelesidis, 2014). A similar feature to the *S. aureus* lpl cluster is the antigenic variation in borrelias resulting from recombination of variable large and small genes (Vidal et al., 2002), and causing the diversity of Vmp lipoproteins involved in immune evasion (Haake, 2000; Schröder et al., 2008).

**Figure 6:**
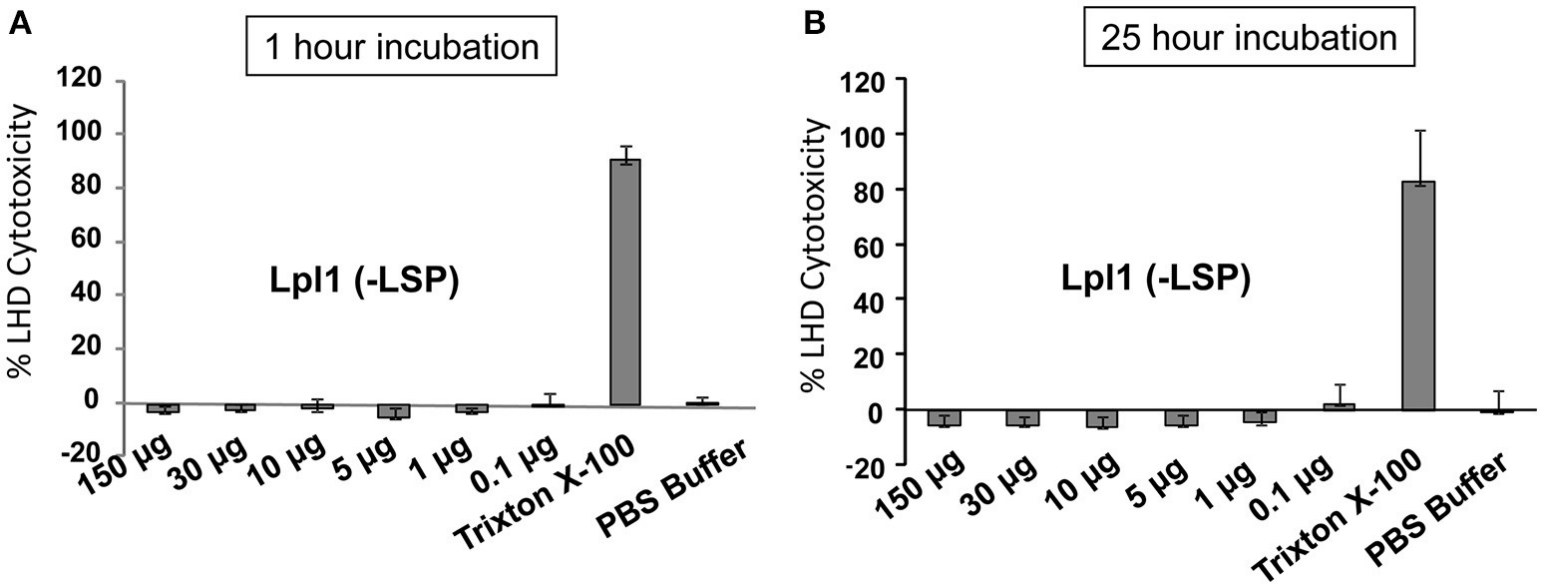
**LDH cytotoxicity assay with purified Lpl1(−LSP) on HaCaT cells**. Various concentrations of purified Lpl1(−LSP) were applied to HaCaT cells for 1 and **(A)** and 25 h **(B)** and the release of LDH into the supernatant was determined. Trixton X-100 (PBS buffer with 0,25% Trixton X-100) and PBS buffer were used as positive and negative controls. Percentage of cytotoxicity was calculated from the difference between treated and control cells.

Our results imply that Lpl1, and most likely also the related Lpl2-Lpl9 lipoproteins, behaves like a bacterial cyclomodulin. This is a new and so far not a described feature of Lpls'. It should be noted that there are a number of differences between Lpl and the other staphylococcal cyclomodulins. All the other known staphylococcal cyclomodulins represent toxins that either disrupt the host membrane or have necrotizing effects. Lpl1 shows no toxic activity with HaCaT cells even at high concentrations (150 μg/ml) and 25 h incubation (Figure 6). Unlike the other cyclomodulins, which are all secreted, Lpls' are anchored in the cytoplasmic membrane with the protein portion facing to the cell wall lumen and beyond. Fixed at the bacteria's cell envelope they are prone to interact with a target protein at the host cells to trigger invasion and G2/M phase transition delay.

## Conclusion

Lpp represent a major class of surface proteins particularly in Gram-positive bacteria. In *S. aureus* they play a role in both nutritional acquisition and in pathogenicity (Shahmirzadi et al., 2016). In staphylococci Lpp are the main TLR2 (toll-like receptor 2) agonists and as such they contribute to innate and adaptive immune signaling, and modulating the immune response and inflammation (Nguyen and Götz, 2016). For immune signaling the lipid modification is absolutely necessary as unlipidated Lpp show no activity. Lpls' represent a specific class of *Staphylococcus aureus* Lpp. Their exact activity is unknown. However, it has been shown that the *lpl* cluster increases immune signaling, host cell invasion and pathogenicity, and may contribute to epidemic spreading of certain strains (Nguyen et al., 2015). Here we show a new function: they exert cyclomodulin activity by delaying the G2/M phase transition. For this activity lipidation of the Lpl is dispensable, it is the protein part that shows this activity. As all Lpl proteins share a highly conserved core sequence there might be a common function that is accentuated by their multiplicity in a tandem gene cluster. Now it would be interesting to know whether there is a correlation of G2/M phase transition delay and host cell invasion and whether the one effect evokes the other.

## Author contributions

MTN, FG, and NB designed the study. MTN, MD, MN, YL, and LP performed the experiments. MTN, FG, and NB wrote the manuscript.

## Funding

This work was supported by the Deutsche Forschungsgemeinschaft (DFG: TRR 34 and SFB 766) and by the French National Institute for Agricultural Research (INRA), GiSA Ruminflame P10552 and Open Access Publishing Fund of Tuebingen University.

### Conflict of interest statement

The authors declare that the research was conducted in the absence of any commercial or financial relationships that could be construed as a potential conflict of interest.

## Supplementary material

Figure S1 **Schematic illustration of the νSaα island, the Δ*lpl* deletion mutant, lpl1 expression plasmids and SDS-PAGE. (A)** Genetic organization of the exotoxin (*set*) and lipoprotein (*lpl*) cluster of νSaα in USA300. **(B)** Creation of USA300Δ*lpl*. **(C)** pTX30::*lpl1*-his is the xylose-inducible expression plasmid for the *lpl1* with the 3' histidine tail and a stop codon. **(D)** pTX30::*lpl1*(−LSP)-his is the xylose-inducible expression plasmid for *lpl*1 without signal peptide with the 3' histidine tail and a stop codon. **(E)** SDS-PAGE of Ni-NTA purified Lpl1(+LSP) and Lpl1(−LSP).

Figure S2 **HPLC analysis of PSM peptides. (A)** HPLC peaks for standard peptides of PSM-α, -β, and -δ (delta toxin). **(B)** PSM peptides in the supernatants of USA300, its Δ*lpl* mutant, and the pTX30-*lpl* complemented mutant. Bacteria were grown aerobically overnight in TSB medium at 37°C.

## Abbreviations

CDK: cyclin-dependent kinases
EDIN: epidermal cell differentiation inhibitor toxin
Hla: staphylococcal alpha toxin
Lpp: Lipoprotein(s)
*lpl*: “*l*ipo*p*rotein-*l*ike” genes
Lpl: lipoprotein-like lipoprotein
*lgt*: diacylglyceryl transferase enzyme encoding gene involved in lipidation of lipoproteins
LSP: lipoprotein signal peptide
Pam2C: (Pam_2_CSK_4_) synthetic dipalmitoylated lipopeptide that mimicks the acylated amino terminus of bacterial lipoproteins
Pam3C: (Pam_3_CSK_4_) synthetic tripalmitoylated lipopeptide
PSM: phenol soluble module.
