# Supplementary figures and images for: Staphylococcus aureus Lpl Lipoproteins Delay G2/M Phase Transition in HeLa Cells
Source: Front Cell Infect Microbiol. 2016 Dec 27;6:201. doi: 10.3389/fcimb.2016.00201 (PMC5187369; doi:10.3389/fcimb.2016.00201)

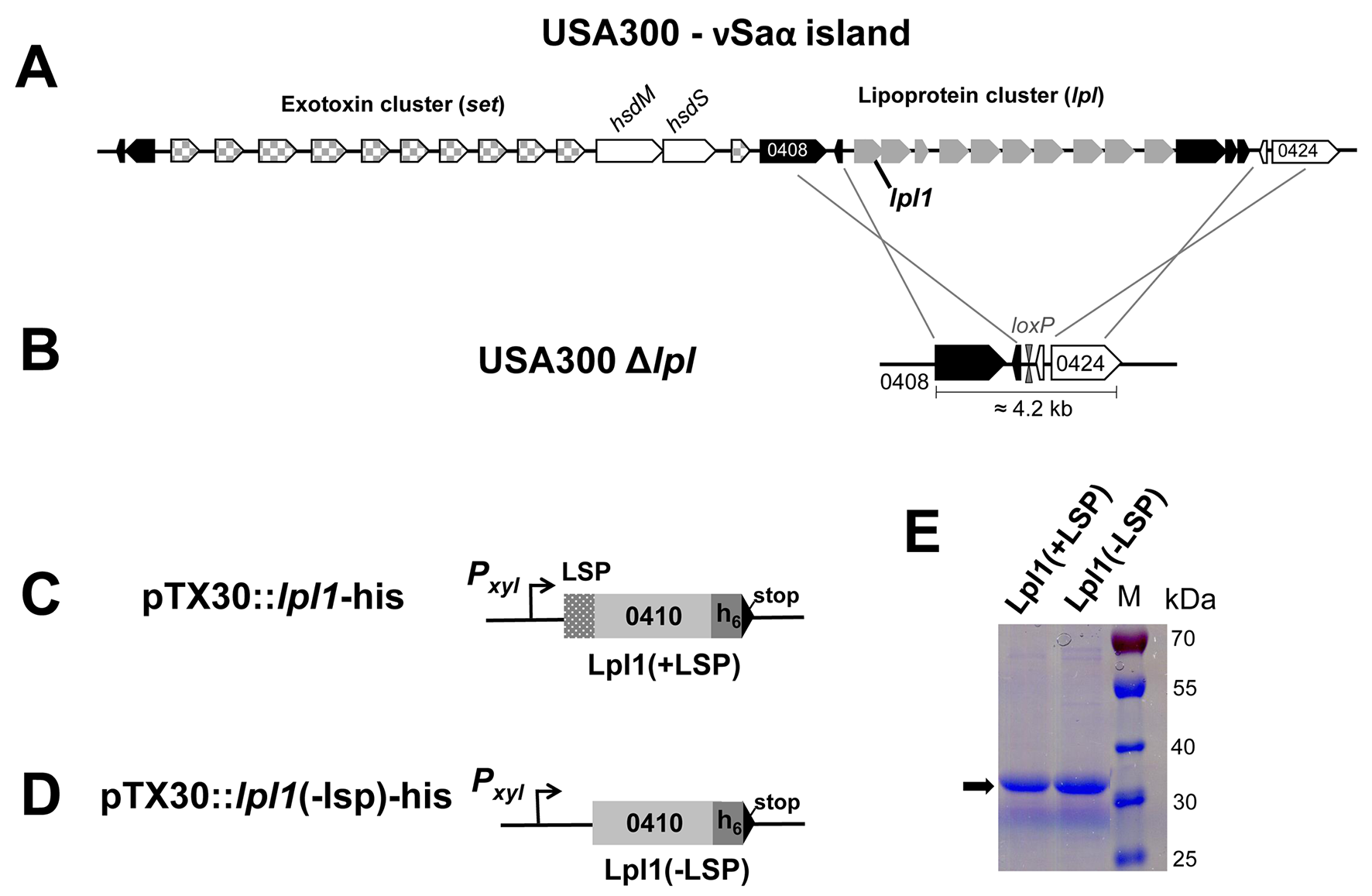

Supplement: Figure S1 — Schematic illustration of the νSaα island, the Δlpl deletion mutant, lpl1 expression plasmids and SDS-PAGE. (A) Genetic organization of the exotoxin (set) and lipoprotein (lpl) cluster of νSaα in USA300. (B) Creation of USA300Δlpl. (C) pTX30::lpl1-his is the xylose-inducible expression plasmid for the lpl1 with the 3' histidine tail and a stop codon. (D) pTX30::lpl1(−LSP)-his is the xylose-inducible expression plasmid for lpl1 without signal peptide with the 3' histidine tail and a stop codon. (E) SDS-PAGE of Ni-NTA purified Lpl1(+LSP) and Lpl1(−LSP). [file Image1.TIF]

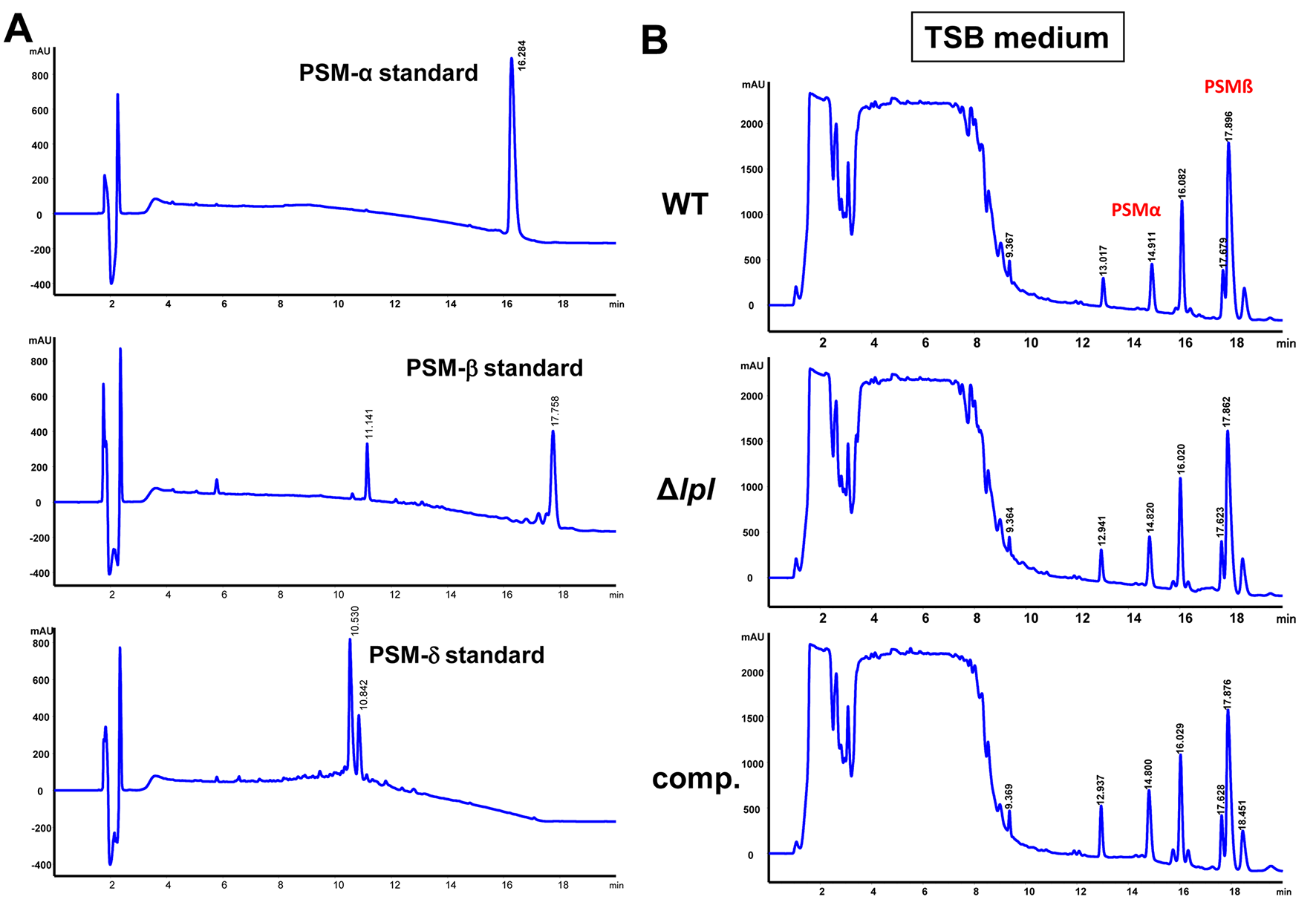

Supplement: Figure S2 — HPLC analysis of PSM peptides. (A) HPLC peaks for standard peptides of PSM-α, -β, and -δ (delta toxin). (B) PSM peptides in the supernatants of USA300, its Δlpl mutant, and the pTX30-lpl complemented mutant. Bacteria were grown aerobically overnight in TSB medium at 37°C. [file Image2.TIF]
